# Circulating NETs enable early identification of thrombotic risk in sepsis at emergency care onset

**DOI:** 10.3389/fimmu.2025.1664108

**Published:** 2025-10-28

**Authors:** Sofia Tejada, Antonio Clemente, Antonia Socias, Maria Aranda, Alberto del Castillo, Joana Mena, Joana Mª Ribas, Luisa Martín, Karla Milagritos Llerena, María Magdalena Arellano, Miguel Agudo, Roberto de la Rica, Marcio Borges

**Affiliations:** ^1^ Multidisciplinary Sepsis Group, Health Research Institute of the Balearic Islands (IdISBa), Palma, Spain; ^2^ Group of Innovation in Immunopathology of Infections (GTERi), Health Research Institute of the Balearic Islands (IdISBa), Palma, Spain; ^3^ Centro de Investigación Biomédica en Red de Enfermedades Infecciosas (CIBERINFEC), Instituto de Salud Carlos III, Madrid, Spain; ^4^ Multidisciplinary Sepsis Unit, Son Llàtzer University Hospital, Palma, Spain; ^5^ Emergency Department, Son Llàtzer University Hospital, Palma, Spain

**Keywords:** sepsis, coagulopathy, NETosis, plasma NETs, biomarkers, emergency department

## Abstract

**Introduction:**

Sepsis involves a dysregulated host response to infection and is frequently complicated by coagulopathy, contributing to organ dysfunction and mortality. Early detection of coagulation disturbances in the emergency department (ED) remains clinically challenging. Neutrophil extracellular traps (NETs) have emerged as key mediators linking inflammation and thrombosis in sepsis, yet their prognostic value during early care is unclear. This study aimed to assess whether circulating NETs measured at sepsis onset are associated with inflammatory biomarkers, sepsis-induced coagulopathy (SIC) status, and clinical outcomes.

**Methods:**

We conducted a retrospective study including 212 adult patients with sepsis recruited at the ED presentation. Plasma NETs, IL-6, and MR-ProADM were measured by ELISA. The ISTH SIC score was used to identify early-stage coagulopathy.

**Results:**

Circulating NETs were detected in 61 patients (28.8%) and higher NETs levels were significantly associated with elevated D-dimer, LDH, IL-6, PCT, and hypocholesterolemia. NETs positive patients had increased odds of positive blood cultures (OR = 2.3; 95% CI: 1.2–2.5), thromboembolic events (OR = 4.4; 95% CI: 1.0–19.0), and SOFA ≥ 5 (OR = 2.0; 95% CI: 1.1–2.9). Among 202 patients with complete data to SIC evaluation, 49% met SIC criteria. Although NETs were not independently associated with SIC, their inflammatory and clinical impact was significantly amplified in SIC-positive patients, suggesting a synergetic interaction between NETosis and early coagulopathy.

**Discussion:**

NETs quantification at ED presentation may help identify a high-risk immunothrombotic phenotype in sepsis and support earlier NETosis-targeted therapies alongside anticoagulation.

## 1 Introduction

Sepsis is a life-threatening condition characterized by a dysregulated host response to infection, leading to acute organ dysfunction and a high risk of death (1). Early diagnosis and timely intervention are essential to improving prognosis (2), particularly in emergency department (ED), where most sepsis cases are initially evaluated (3, 4). However, the clinical manifestations of sepsis are heterogeneous and influenced by the site of infection, the type of pathogen, and patient-related factors such as age and comorbidities (5, 6). Furthermore, sepsis is a biphasic disease that rapidly progress from phases of enhanced inflammation to immune suppression (7). Beyond systemic inflammation, one of the hallmark features of sepsis is a profound disruption of the coagulation system. This may range from subtle endothelial dysfunction to overt sepsis-induced coagulopathy (SIC) or disseminated intravascular coagulation (DIC) (8–10). Early hemostatic alterations often go unrecognized in clinical practice, despite their close association with multiorgan failure, thrombotic complications, and poor outcomes. Early identification of thrombotic risk in the ED remains a major clinical challenge due to the non-specific symptoms of thrombotic events and the limitations of current biomarkers as D-dimer or fibrinogen, which lack specificity (11–13). Thrombotic risk assessment becomes even more complex in sepsis, where inflammation, coagulation abnormalities, and organ dysfunction are deeply intertwined, leading to dynamic and unpredictable changes in hemostasis (8, 14).

One of the key mechanisms linking infection, inflammation, and coagulation in sepsis is the release of neutrophil extracellular traps (NETs) (15). These structures, composed of DNA and proteins, are expelled from neutrophils in response to infection and act as antimicrobial meshes (16). However, excessive or dysregulated release of NETs during NETosis has been implicated in the pathogenesis of sepsis-associated thrombosis (17–19). Components of NETs, such as extracellular DNA and histones, can directly activate the coagulation cascade, promote platelet aggregation, and inhibit fibrinolysis, thereby contributing to the development of SIC and increasing the risk of thromboembolic events (20). Despite strong mechanistic evidence from experimental models, the clinical relevance of plasma NETs as early indicators of hemostatic imbalance in sepsis remains poorly defined (20). Although several studies have explored the role of NETs in critical illness and inflammatory conditions, there is limited evidence regarding their diagnostic or prognostic utility in septic patients during their initial management in the ED (19–22). Some studies have reported that NETosis is associated with inflammatory biomarkers such as interleukin-6 (IL-6) and procalcitonin (PCT), dysregulated coagulation responses (e.g., increased D-dimer, prolonged prothrombin time, and thrombocytopenia) and tissue damage (e.g LDH) in patients with sepsis (23–27). However, these investigations have been conducted mainly in intensive care units (ICUs) or during later stages of the disease. Consequently, it is still unclear whether plasma NETs levels measured at ED presentation correlate with established clinical markers of SIC and can aid in identifying an early prothrombotic phenotype. This gap represents a critical limitation in our current understanding of the pathophysiological connections between innate immunity, vascular homeostasis, and dysregulated coagulation during sepsis.

In this study, we aimed to evaluate the presence and clinical significance of circulating NETs in adult septic patients upon ED admission. Specifically, we analyzed their association with laboratory coagulation parameters, levels of inflammatory and vascular injury biomarkers, organ dysfunction scores, and early clinical outcomes. We also explored the potential modulatory effect of SIC status on these associations. Our goal was to determine whether early NETs detection could serve as a useful marker of SIC and systemic inflammatory dysregulation during the initial phases of hospital care.

## 2 Materials and methods

### 2.1 Study design

A retrospective study was conducted at Son Llàtzer University Hospital in the Balearic Islands (Spain) from December 2021 to July 2023. Adult patients admitted to the ED presenting with clinical suspicion of sepsis were enrolled following activation of the institutional sepsis code protocol and once the diagnosis was confirmed. Sepsis and septic shock definitions were established according to the Third International Consensus Definition (Sepsis-3 Criteria) (1). Each case, whether it met the Sepsis-3 criteria or not, was supervised by expert physicians from a specific sepsis unit at the recruitment hospital. This reinforces the idea that patients were more accurately classified by considering scales such as Sepsis-3, along with individualized criteria like microbiological, clinical, and radiological findings consistent with sepsis.

### 2.2 Data collection

Vital signs assessed during triage were documented at the time of sampling in the ED. Laboratory tests commonly used in the routine protocol for sepsis diagnosis were also collected, including C-reactive protein (CRP), PCT, and lactate levels. Additionally, baseline anticoagulant/antiplatelet therapies, along with clinical and laboratory parameters required to calculate the baseline SOFA score (including coagulation markers, respiratory function, renal profile, and liver function) were systematically collected. For missing values, the next recorded value within the first 24 hours was used. Furthermore, blood culture test results, ICU admission, occurrence of thrombotic events, length of hospitalization, and in-hospital mortality were documented. All of this data is presented in **Table 1**. Sepsis-induced coagulopathy (SIC) was identified according to the SIC score proposed by the International Society on Thrombosis and Hemostasis (ISTH), which includes platelet count, international normalized ratio (INR), and SOFA score (9). A score ≥4 was considered diagnostic for SIC.

**Table 1:** Baseline characteristics, clinical parameters, and biomarker levels in septic patients stratified by circulating NETs levels.

	NETs neg (n=151)	NETs <P50 (n=31)	NETs >P50 (n=30)	P-value
Age, mean (SD)	69.7 (15.2)	71.7 (13.9)	69 (14.4)	0.7914
Sex, n (%)				
Male	100 (66.2)	20 (64.5)	21 (70)	0.8937
Female	51 (33.8)	11 (35.4)	9 (30)	
Symptomatology, n (%)				
> 24 hours	54 (35.8)	9 (29)	7 (23.3)	0.4058
≤ 24 hours	34 (22.5)	11 (35.4)	7 (23.3)	
Unknown	63 (41.7)	12 (38.7)	16 (53.3)	
Triage at ED, n (%)				
2	110 (72.8)	26 (83.9)	20 (66.7)	0.2911
3	41 (27.2)	5 (16.1)	10 (33.3)	
Baseline anticoagulant/antiplatelet therapies, n (%)				
Yes	22 (14.6)	3 (9.7)	7 (23.3)	0.3150
No	128 (84.8)	28 (90.3)	23 (76.7)	
Unknown	1 (0.7)	0 (0)	0 (0)	
Vital signs at ED, median [IQR]				
Heart rate, beats/min	102 [85.7-120]	97 [88-112.5]	107 [91.5-121]	0.4245
Breathing rate, breaths/min	20 [16-26]	19 [16-28]	20 [16.2-27]	0.9686
O2-Saturation, %	96 [93-98]	96 [94-98]	96 [94-97]	0.9306
Body temperature, °C	36.7 [36.3-37.7]	36.4 [36-37.5]	36.9 [36-37.8]	0.4443
Systolic blood pressure, mmHg	109 [97-128]	119 [96-128]	105.5 [88-129.5]	0.4364
Diastolic blood pressure, mmHg	63 [52-74]	66 [57-75]	61.5 [47.7-74.5]	0.5953
Hematological and coagulation profile, median [IQR]				
White blood cell count, x10^9^/L	13.2 [7.3-18.7]	15.6 [8-19.8]	17 [7.2-19.4]	0.4492
Platelets, x10^9^/L	210 [147-319]	235 [159-324]	173 [106.5-259]	0.0921
D-dimer, mg/dL	654 [350-1387]	882 [561.8-1645]	3150 [1030-3837]	**0.0346^#^**
Fibrinogen, mg/dL	823 [665-983]	874 [678-968.5]	844 [702.8-986.5]	0.9898
Prothrombin time, seconds	14.3 [13-17.5]	14.6 [12.7-16.9]	14 [12.8-16.4]	0.6216
Cephaline time, mg/dL	29.3 [26.5-32.8]	29.2 [27-33.8]	29.2 [27.1-33.2]	0.9650
INR	1.3 [1.2-1.6]	1.3 [1.2-1.5]	1.3 [1.2-1.5]	0.9023
APTT, seconds	30.1 [27.4-33.1]	29.1 [26.8-33.8]	28.9 [27-33.1]	0.7411
Biochemistry profile, median [IQR]				
Bilirubin, mg/dL	0.8 [0.5-1.5]	0.8 [0.4-2.3]	0.9 [0.5-1.9]	0.6198
Creatinine, mg/dL	1.1 [0.8-1.8]	1.3 [0.8-1.8]	1.5 [0.9-1.9]	0.4060
Total protein, g/dL	5.7 [5.1-6.4]	6.1 [5.2-6.8]	5.7 [5.4-6.1]	0.4355
Glucose, mg/dL	124 [103-169]	143 [99-174]	115.5 [96-147]	0.5461
LDH, U/L	218 [160-278]	256 [213-539.8]	296 [246-364]	0.0004^*#^
Cholesterol, mg/dL	109 [93-133]	113 [98-144]	85 [79-102]	**0.0099^#±^**
SOFA score, median [IQR]	4 [3-6]	4 [3-6.5]	5 [3.5-7]	0.214
Hospital admission, n (%)	144 (95.3)	28 (90.3)	29 (96.7)	0.4553
Overall length of stay (days)^1^, median [IQR]	6 [4-11]	7 [4-18]	9 [4.5-20]	0.1050
Overall mortality, n (%)	16 (10.6)	4 (12.9)	1 (3.3)	0.4468
Inflammatory biomarkers at ED sampling, median [IQR]				
C-reactive protein, mg/L	22.9 [12.4-39.4]	96.1 [28-224.3]	48 [16.2-262]	0.0002^*#^
PCT, ng/mL	4.3 [1.1-13.4]	2 [0.2-4.4]	17 [3.8-38.4]	**0.0004^#±^**
Lactate, mmol/L	1.7 [1.2-2.8]	1.7 [1.2-3.1]	2.5 [1.6-3.4]	0.0777
IL-6, pg/mL	172 [47-920]	143.6 [43.9-806.7]	399.4 [116.6-8798]	**0.0430^±^**
MR-ProADM, pmol/L	595.7 [301.6-1436]	716.8 [445.5-1238]	963 [415.9-3790]	0.0575

Continuous variables were expressed as mean (SD) or median (IQR), and analyzed using Kruskal-Wallis test. Then, *post-hoc* pairwise comparisons between the three NETs groups were performed using Dunn’s multiple comparisons test. Statistically significant differences are indicated as follows: *NETs neg *vs.* NETs P<50; ^#^NETs neg *vs*. NETs P>50; ^±^NETs P<50 *vs.* NETs P>50. Categorical variables were expressed as frequencies and percentages, and analyzed using contingency tables with Fisher’s exact test. APTT, activated partial thromboplastin time; ED, emergency department; IL-6, interleukin 6; INR, international normalized ratio; IQR, interquartile range; LDH, Lactate dehydrogenase; MR-proADM, Mid-Regional Pro-Adrenomedullin; NETs, Neutrophil Extracellular Traps; PCT, procalcitonine; SD, standard deviation; SOFA, Sequential Organ Failure Assessment. *days from emergency department to discharge for survivors.Statistically significant results are shown in bold (p < 0.05).

### 2.3 Measurement of circulating NETs, IL-6, and MR-ProADM in plasma samples

Blood samples were collected in EDTA vacuum tubes at ED admission. Platelet-free plasma was obtained by double centrifugation and stored at −80°C in the IdISBa Biobank until analysis. Circulating NETs, IL-6 and mid-regional pro-adrenomedullin (MR-ProADM) levels were measured. Circulating NETs were detected in duplicate following a modified in-house ELISA protocol previously reported (28). Briefly, 96-well microplates optimized to bind high amounts of IgG (MaxiSorp trademark from Thermo Fisher Scientific) were coated with rabbit monoclonal antibody to citrullinated histone-3 (abR8Cit-1c from Abcam) at a concentration of 5 μg/mL overnight at 4°C. After three washes using PBS with 0.05% Tween (PBST) microplates were blocked with 300 μL PBS supplemented with 2% BSA per well for two hours at 37°C. During this blocking step, calibration standards were prepared by using the NETs-associated nucleosomes H3R2,8,17Cit dNucs (EpiCypher) in a two-fold dilution series at 2000, 1000, 500, 250, 125, 62.5, 31.3, 15.6, and 0 ng/mL in standard diluent (50 mmol/L Tris-HCl pH 7.5, 300 mmol/L NaCl, 0.01% [w/v] BSA, 0.01% [v/v] Tween-20). Then, plates were washed three times with PBST and incubated at room temperature (RT) for 2 hours following the addition of 20 μL of plasma sample or calibration standard, along with 80 μL of mouse monoclonal detection antibody to DNA at a concentration of 5 μg/mL (Clone 4E9 from Cayman) in standard diluent. After washing, 100 μL of highly cross-adsorbed goat anti-mouse antibody-HRP at 0.1 μg/mL in PBST with 1% BSA was added for 1 hour at RT followed by three washes with PBST. Then, 100 μL of HRP substrate (1-step Ultra TMB from Thermo Scientific) was added and incubated for 15 minutes at RT. Finally the chromogenic reaction was stopped by adding 100 μL of H2SO4 2N and optical density was measured at 450 nm with a PowerWave HT automatic plate reader (Byotek).

IL-6 and MR-ProADM levels were detected in duplicate using a Human PCT sandwich ELISA from Invitrogen (detection range 0.01–20 ng/mL), a Human IL-6 sandwich ELISA from Invitrogen (detection range 7.8–2500 pg/mL) and a Human MR-ProADM sandwich ELISA from Krishgen BioSystems (detection range 15.6–1000 pmol/L), following the manufacturers’ instructions.

### 2.4 Data analysis

Statistical analyses were performed using GraphPad Prism 10. Continuous variables are presented as means and standard deviations (SD) or medians with interquartile ranges (IQR). In some analysis, patients were stratified into three groups: NETs-negative (undetectable NETs), NETs-positive with levels below the median (NETs <P50), NETs-positive with levels above the median (NETs >P50), using the 50th percentile calculated from NETs measurements within the NETs-positive patients (P50 = 112.3 ng/mL) as a cut-off. Comparisons between two or three groups were made using Mann-Whitney tests and Kruskall-Wallis tests, respectively. In the case of Kruskal-Wallis tests, *post-hoc* pairwise comparisons between the three NETs groups (NETs neg, NETs P<50, NETs P>50) were performed using Dunn’s test. Categorical variables were expressed as frequencies and percentages, and analyzed using contingency tables with Fisher’s exact test, accompanied by odds ratios (OR) and 95% confidence intervals (CI). A p-value of ≤0.05 was considered statistically significant.

## 3 Results

### 3.1 Patient characteristics

A total of 212 patients with sepsis were enrolled (33.5% females, mean age 69.8 ± 15.7 years). Septic shock was diagnosed in 18 patients (8.5%) and the overall mortality rate was 9.9%. Blood cultures test were performed in 204 patients (96%), yielding positive result in 68 cases (33.3%). Thirty-seven patients (17.4%) required admission to the ICU, while the remaining patients were admitted to general hospital wards. Thrombotic events occurred in 8 out of 212 patients (3.8%), including 2 cases of deep vein thrombosis (DVT) and 6 cases of pulmonary thromboembolism (PTE). **Table 1** shows all recorded variables in sepsis patients, who were stratified according to circulating NETs levels. After the diagnosis of sepsis and sample collection, 164 patients (77.3%) received prophylactic therapies to prevent thrombosis, including anticoagulants (n=142) or antiplatelet agents (n=22).

### 3.2 Circulating NETs and their relationship with coagulation parameters, biochemistry profile and inflammatory biomarkers

In this study, we used a modified in-house sandwich ELISA for circulating NETs employing a mouse monoclonal antibody (clone 4E9 purchased from Cayman) that specifically targets DNA within NET-associated nucleosomes. Among the anti-DNA antibodies evaluated, only clone 4E9 effectively recognized immobilized nucleosomes in a direct ELISA format (**Supplementary Figure S1**), validating its selection as the detection antibody in our platform. Clone 4E9 was able to replicate the sensitivity of the previously described sandwich ELISA protocol for circulating NETs quantification, while demonstrating an expanded dynamic detection interval than the original method (28), as shown in **Supplementary Figure S2**. Moreover, the experiments performed in **Supplementary Figure S2B** demonstrate that plasma samples did not generate non-specific signals in our ELISA platform. Using this approach, circulating NETs were detected in 61 out of 212 patients with sepsis (28.8%) at ED presentation.

**Figure 1:**
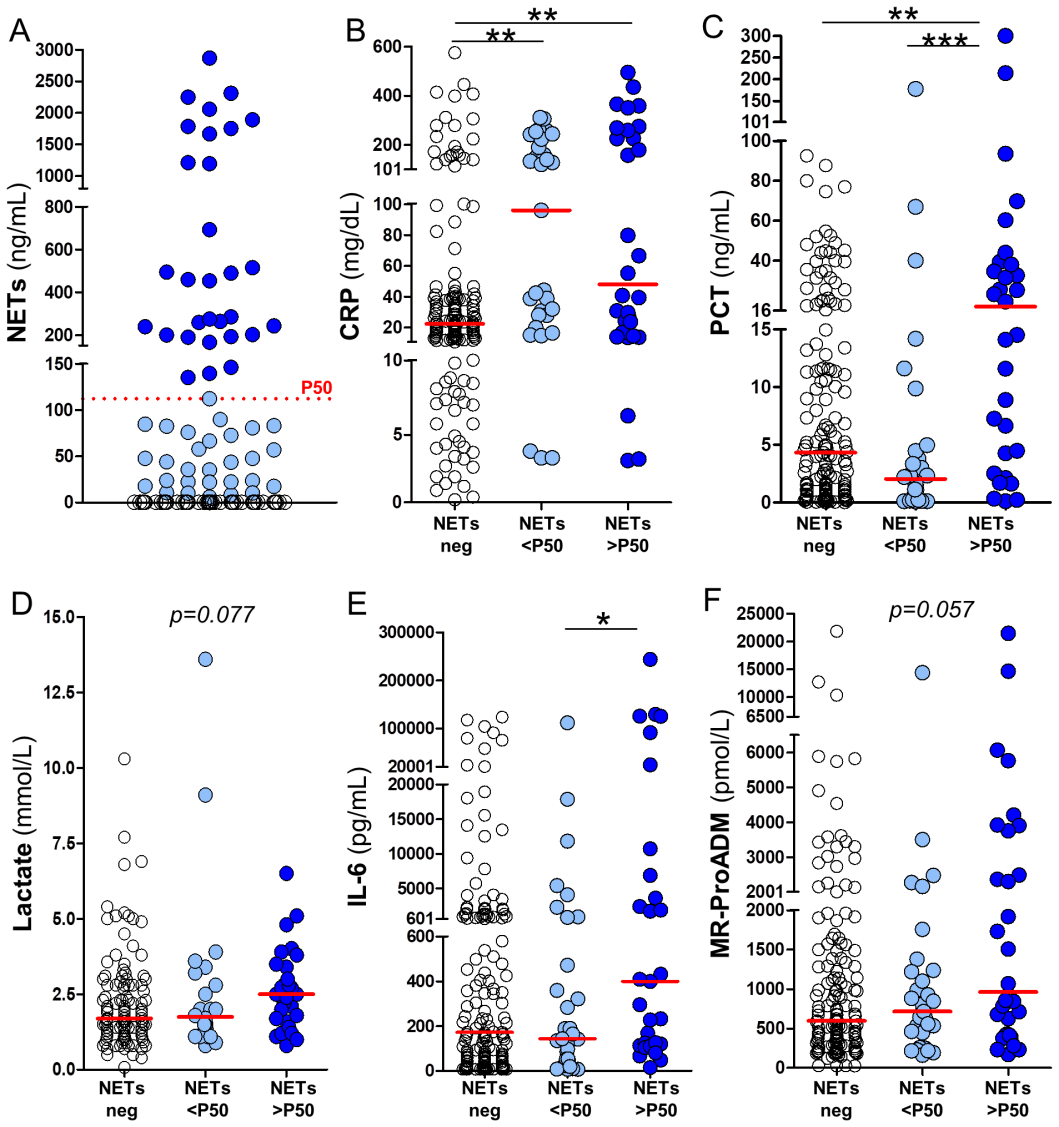
Association between circulating NETs and inflammatory biomarkers in septic patients. **(A)** Patients were stratified based on NETs detection into three groups: NETs-negative (white circles), NETs-positive below the median (NETs <P50; light blue circles), and NETs-positive above the median (NETs >P50; dark blue circles), using the 50th percentile (P50 = 112.3 ng/mL) as the cut-off. Panels **(B–F)** show the plasma levels of inflammatory biomarkers across the different NETs groups in **(A)**, including: **(B)** C-reactive protein (CRP), **(C)** procalcitonin (PCT), **(D)** lactate, **(E)** interleukin-6 (IL-6), and **(F)** mid-regional pro-adrenomedullin (MR-ProADM). Horizontal red lines represent median values. Kruskall–Wallis test *p-*values: *p<0.05, **p<0.01 and ***p < 0.001.

Patients were stratified by NETs levels (**Table 1**) to assess associations between NETosis activation and clinical, laboratory, and outcome variables in sepsis. For analysis, the 50th percentile of circulating NETs levels (P50 = 112.3 ng/mL) was used as a reference threshold. Patients were classified into three groups according to their levels of circulating NETs: no detectable NETs (NETs negative, n=151), NETs levels below P50 as NETs-low (NETs <P50, n=31), and NETs levels above P50 as NETs-high (NETs >P50, n=30). No significant differences were observed among the three patients groups in terms of age, sex, time from symptoms onset to healthcare contact, vital signs at triage or anticoagulant/antiplatelet therapies at baseline. Similarly, the SOFA score, length of hospital stay, and overall mortality rate were comparable among all septic patients, regardless of their circulating NETs levels (**Table 1**). Among the variables included in the hematological and coagulation profile, D-dimer shows a progressive increase across groups stratified by NETs levels (NETs-negative, NETs<P50, and NETs>P50: 654 *vs.* 882 *vs.* 3150 mg/dL, *p* = 0.0346). In the biochemistry profile, higher NETs levels were significantly associated with elevated LDH levels (218 *vs.* 256 *vs*. 296 U/L, *p* = 0.0004), and reduced cholesterol (109 *vs.* 113 *vs*. 85 mg/dL, *p* = 0.0099). We also assessed the relationship between circulating NETs and inflammatory biomarkers commonly used in sepsis management (e.g., CRP, PCT, and lactate), as well as emerging biomarkers such as IL-6 and MR-proADM. A summary of the results concerning these inflammatory biomarkers is provided in **Table 1** and illustrated in **Figure 1**. **Figure 1A** shows the stratification of groups based on quantification of circulating NETs via ELISA. Samples were initially categorized as NETs-negative (empty dots) or NETs-positive (blue dots). NETs-positive samples were further divided based on the P50 threshold (112.3 ng/mL, red dotted line) into low (NETs<P50, light blue dots) and high (NETs>P50, dark blue dots). The same color scheme is applied in **Figures 1B–F** to maintain consistency with the classification established in **Figure 1A**. As expected, plasma levels of all evaluated inflammatory biomarkers were elevated among the septic patients included in the study, regardless of their levels of circulating NETs (**Table 1**; **Figures 1B–F**). However, CRP levels were significantly higher in both groups of septic patients with detectable NETs compared to those with NETs-negative samples (**Figure 1B**, *p*<0.01). PCT levels were significantly higher only in septic patients with circulating NETs>P50 (**Figure 1C**, *p*<0.01 and *p*<0.001), whereas lactate levels showed a similar but non-significant trend (**Figure 1D**, *p*=0.077). Similarly, IL-6 plasma levels were also significantly raised in patients with NETs>P50 (**Figure 1E**, *p*<0.05), and differences in MR-proADM levels nearly reached statistical significance (**Figure 1F**, *p*=0.057). In summary, the results presented in **Table 1** and **Figure 1** indicate that the uncontrolled activation of NETosis during sepsis is associated with changes in hematological and biochemical clinical parameters, as well as with heightened inflammation during early sepsis.

### 3.3 Clinical implications of NETosis activation during sepsis

We constructed contingency tables to assess the association between circulating NETs levels and the occurrence of common clinical complications in sepsis. Contingency analysis in **Table 2** revealed a higher proportion of positive blood cultures in NETs-positive patients compared to NETs-negative patients (45.9% *vs*. 26.5%, *p*=0.009), suggesting a strong association between bacteremia and NETs release. Etiological analysis of bacteremia revealed a predominance of monomicrobial infections (91.2% of positive blood cultures). Among these, *Escherichia coli* emerged as the most frequently isolated pathogen (46.8%), followed by *Klebsiella pneumoniae* and *Streptococcus pneumoniae*, each representing 9.7% of monocrobial infections. A smaller subset of patients presented polymicrobial infections (8.8% of positive blood cultures). Microbial findings from blood cultures are summarized in **Supplementary Table S1**. Altogether, these results not only reinforce the association between NETosis activation and bacteremia, but also emphasize the microbial heterogeneity observed in sepsis. Thromboembolic events were significantly more frequent in the NETs-positive group, occurring in 5 of 61 patients (8.2%), compared to 3 of 151 patients (2.0%) in the NETs-negative group (*p*<0.05). Indeed, the odds of developing thromboembolism were over four times higher in NETs-positive patients (OR=4.4, 95% CI: 1.0-19.0). Additionally, a significantly greater proportion of patients with SOFA scores≥5 were observed in the NETs-positive group compared to the NETs-negative group (60.4% *vs.* 43.0%; *p*=0.036), reflecting a twofold increased risk in the former (OR=2.0, 95% CI: 1.1–3.9). No differences were found between the circulating NETs levels and the incidence of septic shock and ICU admission. Overall, the results in **Table 2** suggest that bacteremia is a key driver of NETosis activation, and that increased circulating NETs are linked to both increased risk of thrombotic events and worsened organ dysfunction in patients with sepsis.

**Table 2:** Clinical implications of activation of NETosis in septic patients.

	NETs-negative (n=151)	NETs-positive (n=61)	Odds ratio (95% CI)	Fisher’s exact test p-value
Negative/Positive blood culture test, n (%)	111 (73.5)/40 (26.5)	33 (54.1)/28 (45.9)	2.3 (1.2-2.5)	**0.0090**
No Septic Shock/Septic Shock, n (%)	141 (93.4)/10 (6.6)	53 (86.9)/8 (13.1)	2.1 (0.8-5.7)	0.1711
No ICU admission/ICU admission, n (%)	126 (83.4)/25 (16.6)	49 (80.3)/12 (19.7)	1.2 (0.6-2.6)	0.6896
No Thrombotic event/Thrombotic event, n (%)	148 (98.0)/3 (2.0)	56 (91.8)/5 (8.2)	4.4 (1.0-19.0)	**0.0457**
SOFA<5/SOFA≥5 (reported in 188 patients), n (%)	77 (57.0)**/58 (43.0)**	21 (39.6)**/32 (60.4)**	2.0 (1.1-3.9)	**0.0358**

CI, confidence interval; ICU, Intensive Care Unit; NETs, Neutrophil Extracellular Traps; OR, Odd Ratio; SOFA, Sequential Organ Failure Assessment. Statistical comparisons were performed using Fisher’s exact test (p<0.05). **Percentages referred to patients with reported SOFA. Statistically significant results are shown in bold (p < 0.05).

### 3.4 Interplay between circulating NETs and sepsis-induced coagulopathy

To explore the relationship between NETosis and early-stage coagulopathy, in **Table 3** we stratified patients according to the ISTH SIC score (9) and compared circulating NETs levels, inflammatory biomarkers, and thrombotic events between SIC-negative (SIC < 4) and SIC-positive (SIC ≥ 4) patients. This study was conducted in 202 patients from our cohort (≈95%) for whom complete data were available to assess SIC. SIC was diagnosed in 99 out of 202 patients (49%), which is consistent with previously reported prevalence in sepsis cohorts (29). As expected, SIC-positive patients had significantly lower platelet counts, higher INR and elevated SOFA scores than SIC-negative patients (*p*<0.001 in all cases, see **Table 3**). These results confirm the clinical relevance of the SIC classification in our settings. The proportion of NETs-positive cases did not differ between SIC groups, nor did the concentration of circulating NETs in samples where NETs were detectable. Nevertheless, PCT and IL-6 plasma levels were more elevated in the group of SIC-positive patients (*p*<0.016 and *p*<0.0004, respectively; see **Table 3**), reflecting a heightened inflammatory response. No significant differences were observed for MR-ProADM, CRP, lactate, or thrombotic event rates.

**Table 3:** Association between SIC diagnosis and NETosis activation, inflammatory biomarkers, and thrombotic events in septic patients.

	SIC <4 (N=103)	SIC ≥4 (N=99)	P-value
Parameters SIC score, median [IQR]			
Platelets, x10^9^/L	253 [181-332]	159 [98.6-267]	**<0.0001**
INR, seconds	1.2 [1.1-1.3]	1.6 [1.4-1.9]	**<0.0001**
SOFA score	4 [2-5]	5 [3-7]	**0.0002**
NETs-negative/NETs-positive, n (%)	73 (70.9)/30 (29.1)	72 (72.7)/27 (27.3)	0.8759
NETs levels in positive samples (ng/mL), median [IQR]	86.5 [32.4-329.2]	139.7 [43.7-454.3]	0.5072
PCT (ng/mL), median [IQR]	2.5 [0.9-11]	6.1 [1.5-21.2]	**0.0116**
IL-6 (pg/mL), median [IQR]	120.8 [28.9-452.9]	357.7 [92.3-2096]	**0.0004**
MR-ProADM (pmol/L), median [IQR]	656.8 [351-1494]	708.6 [312.6-1785]	0.6392
C-reactive protein (mg/L), median [IQR]	26.6 [10.6-100.2]	27.1 [15.1-44.2]	0.6168
Lactate (mmol/L), median [IQR]	1.8 [1.2-2.8]	1.8 [1.2-2.9]	0.6119
Thrombotic event/No thrombotic event, n (%)	5 (4.8)/98 (95.1)	3 (3)/96 (97)	0.7214

SIC, sepsis-induced coagulopathy; IQR, interquartile range; IL-6, interleukin 6; MR-proADM, Mid-Regional Pro-Adrenomedullin; NET, Neutrophil Extracellular Traps; PCT, procalcitonin.Statistically significant results are shown in bold (p < 0.05).

**Figure 2:**
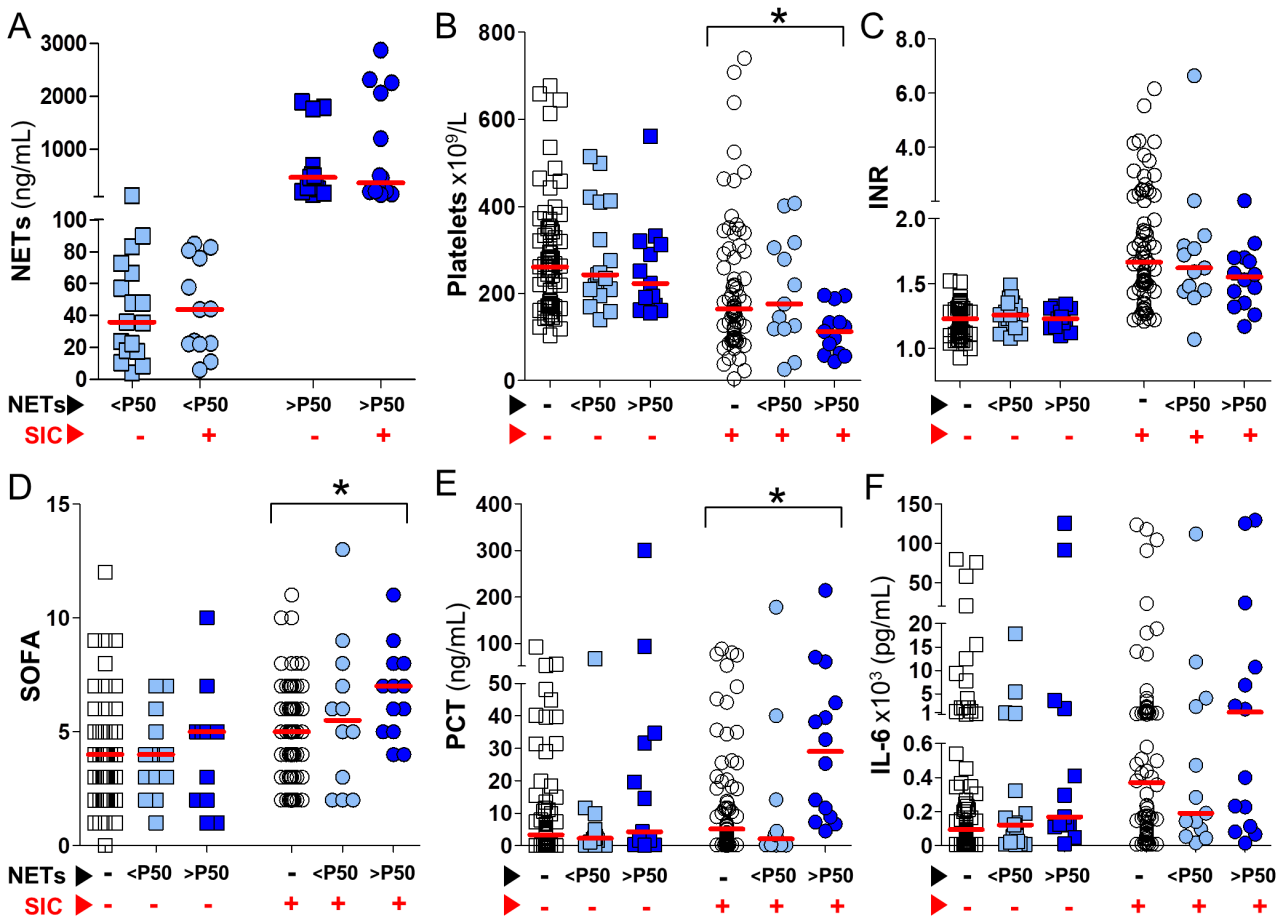
Impact of circulating NETs on SIC-related parameters and inflammatory biomarkers in septic patients. Septic patients were stratified by the presence or absence of SIC according to the ISTH SIC score (red arrows), and further classified into three NETosis activation groups: NETs-negative (white symbols), NETs <P50 (light blue symbols), and NETs >P50 (dark blue symbols), based on circulating NETs levels (black arrows). In all panels, circles represent SIC-positive patients (SIC score ≥4) and squares represent SIC-negative patients (SIC score <4). **(A)** Circulating NETs concentrations in NETs-positive samples from SIC-positive and SIC-negative patients. **(B–F)** Platelet counts, INR, SOFA scores, PCT, and IL-6 levels, plotted according to NETosis activation status in SIC-positive and SIC-negative patients. Comparisons between the three NETosis activation groups were performed using Kruskal–Wallis tests (*p<0.05).

Given the overlap between inflammation and SIC (**Table 3**), and the observation that elevated levels of circulating NETs are associated with increased inflammatory biomarkers (**Figure 1**), we aimed to evaluate in greater detail the interplay between SIC and plasma NETs. To this end, in **Figure 2** patients were first stratified by SIC status (as indicated by red arrows), and subsequently grouped based on their NETs levels (as indicated by black arrows): NETs-negative, or NETs-positive below or above the P50 threshold (white, light blue and dark blue symbols in **Figure 2**, respectively). **Figure 2A** demonstrates that NETs levels in NETs-positive samples, both below and above the P50, were similar between SIC-positive and SIC-negative patients (represented by circles and squares in **Figure 2**, respectively). In **Figure 2B** platelets count decreased as circulating plasma NETs increased, but only in SIC-positive patients (*p*<0.05). The same trend was observed with INR although differences did not reach statistical significance (**Figure 2C**). Interestingly, SOFA scores in **Figure 2D** significantly increased in association with elevated levels of circulating NETs, once again specifically in SIC-positive patients (*p*<0.05). Consistent with this, plasma levels of PCT and IL-6 rose concomitantly with circulating plasma NETs, only in SIC-positive patients (**Figures 2E, F**, respectively), although the differences only reached statistical significance in the case of PCT. Importantly, these changes were not observed in patients without SIC, despite similar NETs concentrations (**Figure 2A**). Altogether, the results presented in **Figure 2** show that while NETs may be activated independently of SIC status, their pathophysiological consequences (i.e., inflammation, endothelial dysfunction, and organ injury) are amplified in the presence of SIC. This supports the role of SIC as a biological amplifier of NET-driven immunothrombosis and highlights the value of integrating NETs quantification with SIC assessment for refined early thrombotic risk stratification in sepsis.

## 4 Discussion

In this study we investigated the activation of NETosis in sepsis patients upon ED admission, and its impact on their clinical condition, with a particular focus on coagulation homeostasis. We demonstrate that circulating NETs are already detectable in nearly 30% of septic patients at ED presentation and are significantly associated with inflammatory activation, coagulation homeostasis, and adverse clinical outcomes. Patients with elevated NETs levels showed higher D-dimer and LDH concentrations, lower cholesterol levels, and greater systemic inflammation as reflected by increased PCT and IL-6. Circulating NETs were also associated with clinically meaningful outcomes such as bacteremia, thromboembolic events, and higher SOFA scores. These associations were present even in the absence of overt coagulopathy, underscoring the potential of NETs to identify a distinct prothrombotic endotype not detected by standard diagnostic tools.

We first standardized a sandwich ELISA for circulating NETs based on a previously reported protocol (28). The capture antibody recognizes citrullinated histone 3, a specific biomarker of PAD4-dependent NETosis, which is a well-established contributor to the pathophysiology of sepsis (30, 31). The detection antibody used in that original protocol is part of a non-separable kit with scarce production details. Here we demonstrate the utility of clone 4E9 in detecting NETs-associated DNA within nucleosomes (**Supplementary Figures S1**, **S2**), offering an alternative detection antibody that is more easily sourced and accompanied by more accessible manufacturing information. This is relevant, because most biomedical studies investigating NETosis in humans focus on detecting isolated NET-associated components, such as citrullinated histones, cell-free DNA, MPO, or neutrophil elastase in circulation, which may lack specificity for true NET formation and function when measured separately (24, 27, 32). Others measure total plasma nucleosomes without addressing specific NETosis biomarkers, which is also not an accurate approach (23). Furthermore, ELISAs to detect MPO-DNA complexes as a measure of NETosis have been somewhat accepted (24, 33), but their use has also been controversial (34). Thus, the optimized ELISA used in this study allowed us to reliably quantify circulating NETs in human plasma (28).

Previous studies have consistently reported that NETosis is activated in sepsis, often in association with inflammatory biomarkers (e.g. plasma CRP, PCT, IL-6 and TNFα), dysregulated coagulation responses (e.g. elevated D-dimer, prolonged prothrombin time and thrombocytopenia) and indicators of tissue damage (e.g. increased LDH) (23–27, 33). Accordingly with these studies, patients in our cohort with higher concentration of circulating plasma NETs exhibited greater levels of CRP, PCT, IL-6, D-dimer and LDH (**Table 1**, **Figure 1**). However, all aforementioned research has focused on patients who were already admitted to hospital wards or ICU settings (23–27). In such contexts it is often challenging to distinguish between early disease mechanisms and consequences of advanced organ dysfunction or treatment interventions. In contrast, our study focuses on the earliest phase of sepsis, prior to significant organ dysfunction or therapeutic interventions (such as prophylactic anticoagulation and fluid resuscitation), allowing a clearer interpretation of NETs as markers of primary pathophysiology. By minimizing the influence of these therapeutic measures known to alter plasma composition and coagulative responses (35, 36), we were able to more accurately assess the potential relationships between NETosis, systemic inflammation, and coagulopathy.

Interestingly, we also report here for the first time that samples with higher levels of circulating plasma NETs were observed in sepsis patients with more severe hypocholesterolemia (**Table 1**). Hypocholesterolemia has previously been associated with poor outcomes in sepsis (37), and experimental data suggest that low cellular cholesterol levels facilitate formation of NETs by neutrophils (38). This raises the intriguing possibility that cholesterol depletion may enhance NETosis during sepsis, linking metabolic dysregulation to exacerbated innate immune activation.

From a clinical perspective, our findings support the potential utility of circulating NETs as early biomarkers to complement current diagnostic tools for stratifying the risk of sepsis-related complications. Unlike traditional inflammatory markers, which may reflect a generalized host response, circulating NETs provide mechanistic insight into the activation of immunothrombosis, a process closely linked to organ dysfunction and vascular complications (39). In this context, our findings reveal a clear association between elevated NETs and increased inflammatory burden (i.e., CRP, PCT, IL-6), markers of coagulation imbalance (i.e., D-dimer), and tissue damage (i.e., LDH) at the time of ED presentation, suggesting that NETs could serve as early indicators of a prothrombotic endotype and multiorgan damage in sepsis. Our investigation also demonstrates that circulating NETs are associated with a higher risk of thromboembolic events and greater organ dysfunction. Previous studies have shown that NETs can act as both structural and functional scaffold for thrombus formation, enhancing platelet activation and thrombin generation (40, 41). Although the link between NETs and coagulopathy has been reported in settings such as trauma and COVID-19 (42–44), clinical studies specifically addressing this relationship in sepsis remain limited. Some studies have emphasized the role of NETosis in microvascular thrombosis and laboratory-defined coagulopathies, such as DIC in ICU settings (26, 45). In contrast, our study provides novel evidence linking circulating NETs to clinically overt thromboembolic events, including PE and DVT, from the very onset of sepsis, at the time of ED presentation. As anticipated, the overall frequency of thromboembolism was 3.8%, a rate consistent with expected values. However, its occurrence was significantly higher in NETs-positive patients, who exhibited more than a fourfold increased risk compared to those without detectable NETs. These findings suggest that NETosis may play an active role in the early development of macrovascular thrombosis during sepsis.

To better interpret these findings in the context of coagulation disturbances, we stratified patients by the presence or absence of SIC, the earliest clinically recognized stage of coagulation dysfunction in sepsis. The SIC score was designed to detect the non-overt phase of DIC, which may progress to overt DIC as the condition worsens. Its clinical utility has been supported by multiple studies since its introduction (29). However, in our cohort, 5 out of 8 thromboembolic events occurred in patients who did not meet SIC criteria, despite all patients receiving prophylactic anticoagulants. This finding underscores a critical limitation of SIC scoring to detect all patients at risk of thrombosis, suggesting that NETs may reflect a distinct prothrombotic endotype that is not fully captured by the current SIC criteria. Thus, incorporating NETs quantification into future diagnostic models could enhance early risk stratification and offer a more comprehensive view of coagulation imbalance in sepsis. Importantly, although NETs levels did not differ significantly between SIC-positive and SIC-negative patients, the clinical impact of circulating NETs was more pronounced in those with SIC, suggesting that NETosis may play a synergistic role in driving disease severity when coagulation pathways are already altered. This suggests that NETs could serve as early markers of immunothrombosis and guide targeted treatments, such as personalized anticoagulation or NET-inhibiting therapies (19), especially for high-risk patients in the ED.

It is important to note that circulating NETs were detectable in 26.5% of patients with negative blood cultures. The detection of NETs in cases without confirmed bacteremia underscores their potential as host-derived biomarkers that do not rely on microbiological confirmation. This is particularly relevant in emergency settings, where blood cultures have limited sensitivity and results are not immediately available. In this context, NETs quantification could provide a valuable support in the early identification of patients at risk of severe sepsis, regardless of culture status.

This study has some limitations. First, it was conducted in a single center with a relatively small sample size, which may limit the generalizability of the findings. Second, NETs and other biomarkers were measured at a single time point, which precludes any analysis of their temporal evolution during sepsis. While longitudinal data could offer additional insights, repeated measurements might also be confounded by subsequent interventions such as anticoagulation or fluid resuscitation. Finally, our ELISA specifically targets PAD4-dependent NETosis, which has been identified as a key contributor to organ damage and immunothrombotic responses during sepsis (30, 31). Alternative NETosis pathways, such as those independent of PAD4, were not assessed in this study. PAD4-independent NETosis is typically mediated by MPO and neutrophil elastase without histone citrullination, and it is more prominent during the initial stages of host defense to neutralize pathogens. Although not directly evaluated, its potential influence on the observed outcomes cannot be ruled out. A major strength of this study resides in its real-world design, enrolling patients during their initial presentation to the ED, an underexplored setting where early biomarkers are most urgently needed to support rapid clinical decisions. In our study, circulating NETs were measured at a laboratory after samples were collected, frozen, and stored until analysis by ELISA. However, for this biomarker to be useful in real-world settings, it must be available in real-time to guide the clinical management of septic patients in the ED settings. Therefore, developing new point-of-care tools for rapid evaluation of NETosis activation is critically important.

In conclusion, circulating NETs are detectable at the very early stages of sepsis, even in the absence of bacteremia or overt coagulopathy, and are consistently associated with inflammatory, hemostatic imbalance, and adverse clinical outcomes. Assessing circulating NETs may help detect high-risk patients with an immunothrombotic phenotype that standard diagnostic criteria fail to recognize, while also providing tailored treatment approaches.

## Data Availability

The data that support the findings of this study are not openly available due to reasons of sensitivity and are available from the corresponding author (AC or MB) upon reasonable request. Data are located in controlled access data storage at Sepsis Unit department of Son Llàtzer University Hospital.
