# Supplementary material for: Circulating NETs enable early identification of thrombotic risk in sepsis at emergency care onset
Source: Front Immunol. 2025 Oct 28;16:1664108. doi: 10.3389/fimmu.2025.1664108 (PMC12602214; doi:10.3389/fimmu.2025.1664108)
Supplement: Supplementary file 1 [file DataSheet1.docx]

Supplementary Material for

**Circulating NETs Enable Early Identification of Thrombotic Risk in Sepsis at Emergency Care Onset**

**Sofía Tejada,^1^ Antonio Clemente,^2,3,*^ Antonia Socias,^1,4^ Maria Aranda,^1,4^ Alberto del Castillo,^1,4^ Joana Mena,^1,4^ Joana Mª Ribas,^1,5^ Luisa Martín,^1,5^ Karla Milagritos Llerena,^1,5^ María Magdalena Arellano,^1,5^ Miguel Agudo,^5^ Roberto de la Rica,^1,3^ and Marcio Borges,^1,4,*^**

^1^ Multidisciplinary Sepsis Group, Health Research Institute of the Balearic Islands (IdISBa), Palma, Spain.

^2^ Group of Innovation in Immunopathology of Infections (GTERi), Health Research Institute of the Balearic Islands (IdISBa), Palma, Spain.

^3^ CIBER de Enfermedades Infecciosas (CIBERINFEC), Instituto de Salud Carlos III Madrid, Spain.

^4^ Multidisciplinary Sepsis Unit, Son Llàtzer University Hospital, Palma, Spain.

^5^ Emergency Department, Son Llàtzer University Hospital, Palma, Spain.

**Table of contents:**

**Section 1.** Direct ELISA for immobilized NETs-associated nucleosomes.

**Supplementary Figure S1.** Calibration curves of NETs detection using a direct ELISA with different antibodies against DNA.

**Section 2.** Validation of the sandwich ELISA for circulating NETs.

**Supplementary Figure S2.** Calibration curve of NETs detection using the modified sandwich ELISA protocol proposed in the study.

**Supplementary Table 1.** Microbial isolates identified from blood cultures in the sepsis cohort.

**Section 1. Direct ELISA for immobilized NETs-associated nucleosomes.**

In this study, we aimed to follow a previously reported protocol for the detection of NETs using a sandwich ELISA (reference 29 in the main text). However, the protocol lacked specific information regarding the detection antibody used against NETs-associated DNA. Additionally, the mentioned detection antibody is part of a kit; therefore, supply issues further impeded the implementation of the described method. We aimed to explore the feasibility of using different anti-DNA antibodies as detection antibodies in the sandwich ELISA for NETs. To this end, we immobilized NETs-associated nucleosomes onto the plate wells by drying solutions containing nucleosomes, which were subsequently detected using the selected detection antibodies in a direct ELISA format. We included three potential detection antibodies: a monoclonal mouse IgG anti-double stranded DNA antibody from Merck Millipore (clone AE-2), a monoclonal mouse IgM anti-DNA antibody from Thermo Scientific (clone ET844.3.1), and a monoclonal mouse IgG anti-DNA antibody from Cayman (clone 4E9). Briefly, 100 μL of samples containing increasing concentrations of NETs in PBS (2000, 1000, 500, 250, 125, 62.5, 31.3, 15.6, and 0 ng/mL) was added to a 96-well ELISA plates and dried by overnight incubation at 37 ºC on a heating plate. Next, plates were washed 3 times with PBS containing 0.1% Tween 20 (PBST 0.1%), blocked during 2 h at room temperature (RT) with PBS containing 5 mg·mL^-1^ bovine serum albumin (BSA) and washed again 3 times with PBST 0.1%. Then, 100 μL of detection antibody anti-DNA at 0.5 μg·mL^-1^ in PBST 0.1% supplemented with 1% BSA was added and incubated for 1 hour at RT. The subsequent steps differed depending on the detection antibody used:

1. For the mouse IgG anti-DNA antibodies; after washing, 100 μL of highly cross-adsorbed and biotin-conjugated goat anti-IgG-mouse antibody (Thermo Scientific) at 0.5 μg/mL in PBST 0.05% with 1% BSA was added for 1 hour at RT, followed by five washes with PBST 0.1%. Next, 100 μL of High sensitive streptavidin-HRP (Thermo Scientific) diluted at 1:200000 in PBST 0.05% with 1% BSA was added for 30 min at RT, followed by five washes with PBST 0.1%. Then, 100 μL of HRP substrate (1-step Ultra TMB from Thermo Scientific) was added and incubated for 15 minutes at RT.
2. For the mouse IgM anti-DNA antibody; after washing, 100 μL of goat polyclonal anti-IgM-mouse antibody-HRP (Abcam) at 0.5 μg/mL in PBST 0.05% with 1% BSA was added for 1 hour at RT followed by five washes with PBST 0.1%.

Finally the chromogenic reaction was stopped by adding 100 μL of H_2_SO_4_ 2N and absorbance was measured at 450 nm with a PowerWave HT plate reader (Byotek).

In supplementary Figure 1, only the mouse monoclonal IgG anti-DNA antibody from Cayman (clone 4E9) yielded increasing absorbance values in a dose-dependent manner (blue line), whereas the other antibodies tested produced no detectable signals, regardless the NETs concentration used (black and red lines). These results validated the use of the mouse monoclonal IgG anti-DNA clone 4E9 as the detection antibody in our in-house sandwich ELISA for circulating NETs.

**
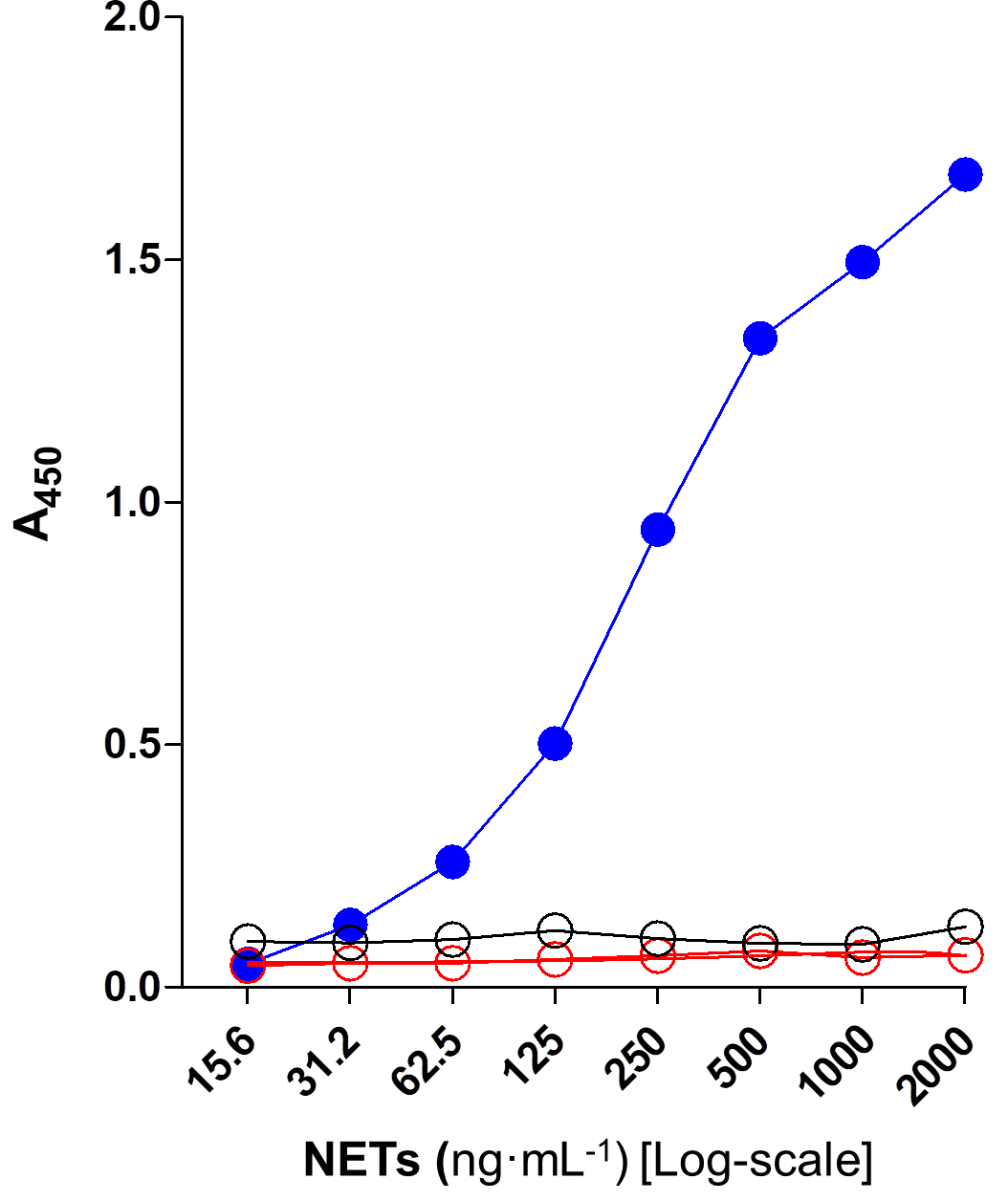
**

**Supplementary Figure 1.** Calibration curves of NETs detection using a direct ELISA with different antibodies against DNA. Dots represent the mean of 3 replicates for each NETs standard concentration, tested with the IgG anti-double stranded DNA antibody clone AE-2 (black dots), the mouse IgM anti-DNA antibody clone ET844.3.1 (red dots), and the mouse monoclonal IgG anti-DNA antibody clone 4E9 (blue dots).

**Section 2.** **Validation of the sandwich ELISA for circulating NETs.**

To validate the protocol of our in-house sandwich ELISA for detecting circulating NETs, we assessed calibration curves across four independent experiments, following the procedure described in the main text. Additionally, we evaluated whether plasma samples could produce non-specific signals in the ELISA due to matrix interferences. To this end, we selected a representative set of plasma samples from sepsis patients included in the study (n = 96, 47.5%) and incubated them in wells lacking the capture antibody.

Supplementary Figure 2A shows the calibration curve obtained from four independent experiments, validating the performance and reproducibility of our platform. Moreover, plasma samples did not generate significant background signals when applied to wells without the capture antibody, indicating that sample matrices do not interfere through non-specific interactions with the detection antibody (Supplementary Figure 2B). In fact, in Supplementary Figure 2B the background signals produced by plasma samples (red bar) were even lower that those by sample diluent buffer (black bar). These results support the specificity of the assay for detecting circulating NETs and eliminate the need to subtract background signals from each plasma sample to estimate the specific signals attributed to circulating NET detection.

**
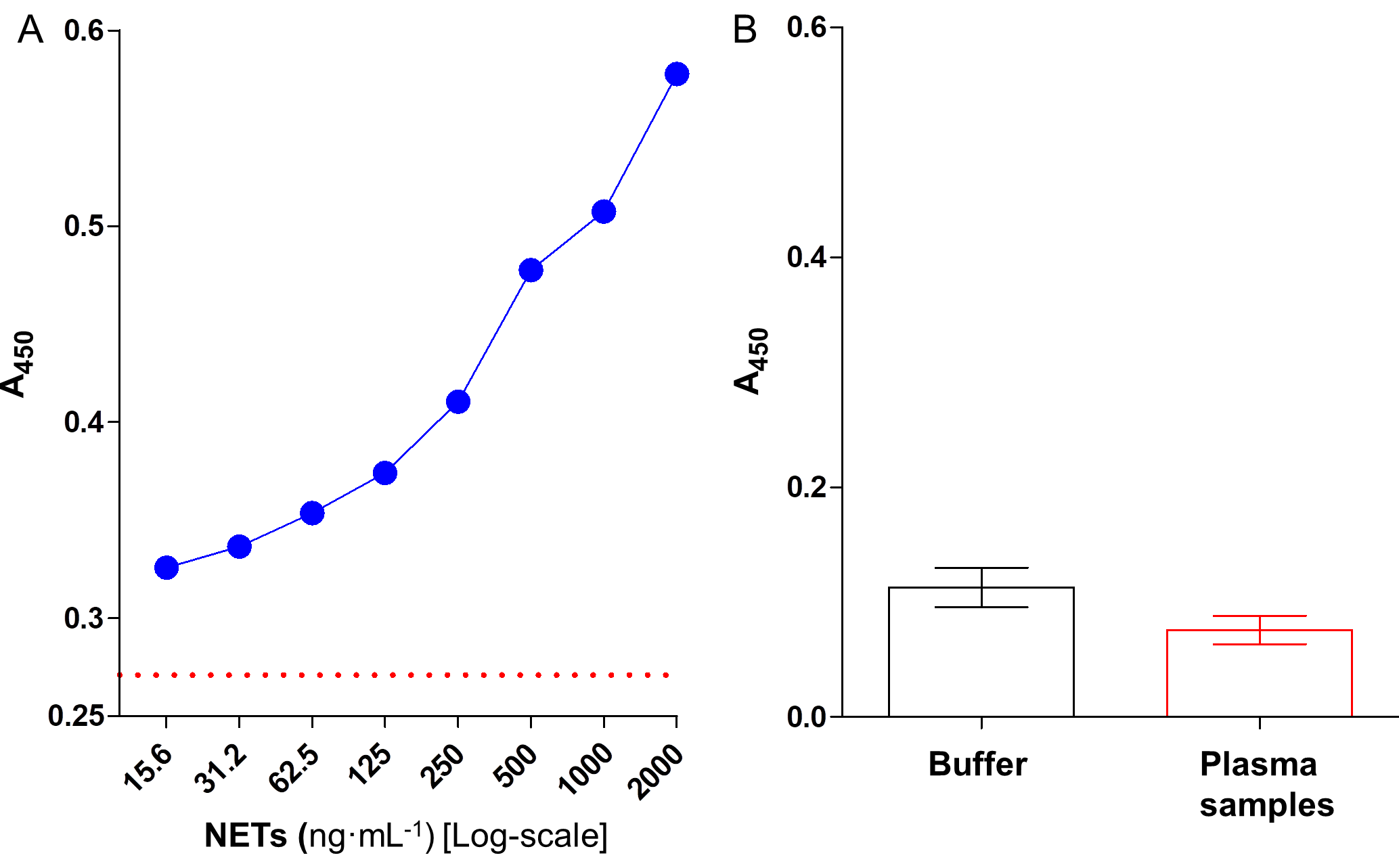
**

**Supplementary Figure S2. (A)** Calibration curve of NETs detection using the modified sandwich ELISA proposed in the study. The assay procedure is detailed in the main text. Method validation was performed by assessing four independent calibration curves. Blue dots represent the average across all independent curves, based on the means of three replicates per concentration in each experiment. The red dotted line indicates the mean + 2 SD of the signal obtained with the standard diluent (0 ng/mL NETs). **(B)** Bars represent de mean of the absorbance signals produced by the sample diluent buffer used in the ELISA (black, n=15) and by plasma samples from sepsis patients included in the study (red, n=96), in the absence of capture antibody.

**Supplementary Table 1. Microbial isolates identified from blood cultures in the sepsis cohort.**

| **Type** | **Pathogen(s)** | **Cases, n (%)** |
| --- | --- | --- |
| **Monomicrobial** | *Escherichia coli* | 29 (46.8) |
| **(n=62)** | *Klebsiella pneumoniae* | 6 (9.8) |
|  | *Streptococcus pneumoniae* | 6 (9.8) |
|  | *Staphylococcus aureus* | 4 (6.4) |
|  | *Haemophilus influenza* | 3 (4.8) |
|  | *Pseudomonas aeruginosa* | 2 (3.2) |
|  | *Staphylococcus epidermidis* | 2 (3.2) |
|  | *Actinomyces neuii* | 1 (1.6) |
|  | *Aeromonas sobria* | 1 (1.6) |
|  | *Proteus mirabilis* | 1 (1.6) |
|  | *Enterobacter aerogenes* | 1 (1.6) |
|  | *Enterobacter cloacae* | 1 (1.6) |
|  | *Morganella morganii* | 1 (1.6) |
|  | *Serratia marcescens* | 1 (1.6) |
|  | *Streptococcus agalactiae* | 1 (1.6) |
|  | *Staphylococcus capitis* | 1 (1.6) |
|  | *Staphylococcus warneri* | 1 (1.6) |
| **Polymicrobial** | *Streptococcus parasanguinis + Veillonella párvula* |  |
| **(n=6)** | *Psychrobacter phenylpyruvicus + Arthrobacter cumminsii + Staphylococcus aureus + Proteus mirabilis* |  |
|  | *Enterococcus faecalis + Escherichia coli + Klebsiella pneumoniae* |  |
|  | *Escherichia coli + Staphylococcus cohnii* |  |
|  | *Escherichia coli + Klebsiella oxytoca* |  |
|  | *Fusobacterium necrophorum + Bacteroides fragilis* |  |
